# Cardiopulmonary adaptations of a diving marine mammal, the bottlenose dolphin: Physiology during anesthesia

**DOI:** 10.14814/phy2.16183

**Published:** 2024-09-08

**Authors:** Carolina R. Le‐Bert, Gordon S. Mitchell, Leah R. Reznikov

**Affiliations:** ^1^ Department of Physiology & Aging, College of Medicine University of Florida Gainesville Florida USA; ^2^ U.S. Navy Marine Mammal Program Naval Information Warfare Center Pacific San Diego California USA; ^3^ Department of Physical Therapy, College of Public Human and Health Professionals University of Florida Gainesville Florida USA; ^4^ Department of Physiological Sciences, College of Veterinary Medicine University of Florida Gainesville Florida USA

**Keywords:** anesthesia, cardiopulmonary, cardiovascular, cetacean, dolphin, perfusion adaptations, physiology, pulmonary, ventilation

## Abstract

Diving marine mammals are a diverse group of semi‐ to completely aquatic species. Some species are targets of conservation and rehabilitation efforts; other populations are permanently housed under human care and may contribute to clinical and biomedical investigations. Veterinary medical care for species under human care, at times, may necessitate the use of general anesthesia for diagnostic and surgical indications. However, the unique physiologic and anatomic adaptations of one representative diving marine mammal, the bottlenose dolphin, present several challenges in providing ventilatory and cardiovascular support to maintain adequate organ perfusion under general anesthesia. The goal of this review is to highlight the unique cardiopulmonary adaptations of the completely aquatic bottlenose dolphin (*Tursiops truncatus*), and to identify knowledge gaps in our understanding of how those adaptations influence their physiology and pose potential challenges for sedation and anesthesia of these mammals.

## Graphical abstract

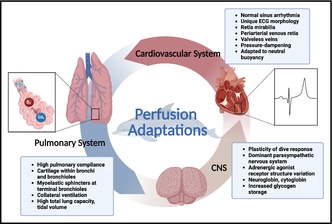

## 1 INTRODUCTION

Approximately 50–55 million years ago, a terrestrial artiodactyl similar to a small deer made the transition from land to water (Thewissen et al., 2009, 2007). Fossil records suggest that this ancestor of cetaceans became more amphibious over millennia until it became fully aquatic. Cetacea is the mammalian infraorder that includes whales, dolphins and porpoises. The anatomic and physiologic modifications of cetaceans likely provided evolutionary advantages to survival in completely aquatic ecosystems (Dolar et al., 1999; Kooyman & Ponganis, 1997; Piscitelli et al., 2010, 2013). Within the suborder of toothed whales (*Odontocetes*), a relatively small, shallow‐diving cetacean, the bottlenose dolphin (*Tursiops truncatus*), is the most extensively studied in its natural environment and while housed under the care of humans. Observational and capture‐release research of wild dolphin populations has provided copious information on dolphin natural history, disease ecology, and diving physiology, as well as historical and current conditions of ocean health (Schwacke et al., 2012; Wells, 2009; Yordy et al., 2010). While housed under human care, bottlenose dolphins often receive comprehensive veterinary medical services and may even contribute to clinical and translational biomedical research (Houser, Finneran, & Ridgway, 2010; Le‐Bert et al., 2018; Meegan, Ardente, et al., 2021; Venn‐Watson et al., 2015, 2022). General anesthesia, however, remains a challenge in dolphins due to a limited number of experienced anesthesiologists and published studies, the significant limitations of current commercially‐available ventilators, and limited anesthetic drug pharmacokinetic studies, including their effects on whole body physiology (Bailey, 2021; Doescher et al., 2018; Dold & Ridgway, 2014; Dover et al., 1999; Higgins & Hendrickson, 2013; Howard et al., 2006; Jones et al., 2023; Le‐Bert et al., 2024; Lee et al., 2019; Lindemann et al., 2023; McCormick & Ridgway, 2018; Medway et al., 1970; Meegan et al., 2015, 2016; Nagel et al., 1964, 1966, 1968; Ridgway, 2002; Ridgway et al., 1975, 1974; Ridgway & McCormick, 1971, 1967; Rosenberg et al., 2017; Russell et al., 2021; Schmitt et al., 2014, 2018; Sommer et al., 1968; Tamura et al., 2017). In this review, we aim to synthesize the current understanding of anesthesia physiology with knowledge of the normal cardiopulmonary physiology and subsequent perfusion adaptations of dolphins and how these adaptations may be modulated during general anesthesia of this completely aquatic marine mammal.

## 2 HISTORY OF DOLPHIN ANESTHESIA

General anesthesia of dolphins is an infrequently practiced discipline within veterinary medicine. Little technical and practical progress was made between the first dolphin to ever be anesthetized in 1932 and the 1960s (Lilly, 1964; Nagel et al., 1964, 1966). However, during the 1960s and 1970s, Ridgway, Nagel, McCormick and colleagues made significant progress in the successful induction of, and emergence from, anesthesia in dolphins (Medway et al., 1970; Nagel et al., 1964, 1966, 1968; Ridgway et al., 1974; Ridgway & McCormick, 1971, 1967; Sommer et al., 1968). During this period of time, induction was often achieved with intravenous barbiturates (i.e., sodium thiopental, 10–25 mg/kg) and a surgical plane of anesthesia maintained with the volatile gas, halothane, or a nitrous oxide‐oxygen mixture (Table 1). Mechanical ventilation was achieved through adaptation of a Bird Mark 9 large animal ventilator (Bird Respirator Company, Palm Springs, CA) with a custom‐designed apneustic plateau control unit, created to mimic the breath‐holding apneustic breathing pattern of cetaceans. Apneustic plateau ventilation, as coined by Ridgway and McCormick, enabled rapid lung inflation with an inspiratory breath hold at approximately 20–24 mmHg pressure for 15–30 s, followed by airway pressure release, and rapid re‐inflation (Ridgway et al., 1974; Ridgway & McCormick, 1971, 1967). Early ventilation practices using conventional modes of ventilation would result in decreasing trends towards hypoxemia due to hypoventilation (Ridgway et al., 1974). Thus, apneustic plateau ventilation was the standardized approach for mechanical ventilation of dolphins.

**TABLE 1:** Summary of general anesthesia performed during anatomic and physiologic studies of dolphins (*Tursiops* spp.)

Study objective(s)	No	Age (years)	Weight range (kg)	Pre‐medication agent(s)	Induction agent(s)	Maintenance agent(s)	MAP range (mmHg)	Reversal agent(s)	Reference
Effect of halothane anesthesia on hepatic damage during auditory research	6 (F = 3, M = 3)	N/A	N/A	N/A	Sodium thiopental, 10–15 mg/kg, IV	Halothane	115	N/A	Medway et al., 1970; Ridgway & McCormick, 1971
Bispectral index monitor to detect interhemispheric asymmetry	3 (F = 1, M = 2)	N/A	212–263	Diazepam, 0.15 mg/kg, PO (*n* = 1)	Propofol, 3.03–4.72 mg/kg, IV	N/A		N/A	Howard et al., 2006
Anesthesia induction and maintenance with thiopental and halothane	10	N/A	N/A	N/A	N/A	Halothane		N/A	Ridgway & McCormick, 1967
	5	N/A	N/A	N/A	Sodium thiopental, 10 mg/kg, IV	Halothane		N/A	
Surgical approach to the dolphin ear	4	N/A	N/A	Atropine, 0.02 mg/kg, IM	Sodium thiopental, 10–15 mg/kg, IV	Halothane, 1–2%		N/A	Ridgway et al., 1974
Hemodynamic and coronary angiographic studies in the dolphin	4	N/A	80–114	N/A	Pentobarbital, 10 mg/kg, IP	Nitrous oxide‐oxygen	122–142	N/A	Sommer et al., 1968; Nagel et al., 1964; Nagel et al., 1966; Nagel et al., 1968
Return of sound production following anesthetic recovery	10 (F = 4, M = 6)	8–46 (mean 32.4)	N/A	Midazolam, 0.08–0.1 mg/kg, IM	Midazolam, 0.02 mg/kg, IV	Sevoflurane	N/A	1:13 (Midazolam: Flumazenil), IV	Jones et al., 2023
				Meperidine, 0.1–0.2 mg/kg, IM	Propofol, 1–4 mg/kg, IV			Naloxone, 0.01 mg/kg, IV	
					cis‐Atracurium, 0.1 mg/kg, IV				
Apneustic anesthesia ventilation on pulmonary function	9 (F = 3, M = 6)	10–42 (mean 32)	141–292	Diazepam, 0.08–0.30 mg/kg, PO (*n* = 2)	Midazolam, 0.02 mg/kg, IV	Sevoflurane, 1.8–2.0%	80.8+/− 2.9; 86+/−2.6	Flumazenil, 0.02–0.05 mg/kg, IM/IV	Le‐Bert et al., 2024
				Midazolam, 0.08–0.1 mg/kg, IM	Propofol, 2–4 mg/kg, IV			Naloxone, 0.01–0.04 mg/kg, IV (*n* = 7)	
				Meperidine, 0.1–0.2 mg/kg, IM				Naltrexone, 0.05–0.20 mg/kg, IV (*n* = 6)	
Plasma propofol concentrations in dolphins	6	12–27 (mean unk)	N/A	Diazepam, PO	Propofol, 1.97–5.33 mg/kg, IV	Sevoflurane	N/A	N/A	Schmitt et al., 2018
				Midazolam, IM	Midazolam, IV				

Anesthetic practices in the 60s and 70s evaluated the use of a low solubility anesthetic gas, nitrous oxide, for maintaining a surgical plane of anesthesia. This minimally potent inhalational anesthetic was often combined with a neuromuscular blocking agent (succinylcholine) and a parenteral barbiturate (thiopental) in dolphins. However, a mixed gas anesthetic protocol of 60% nitrous oxide with 40% oxygen did not result in a surgical plane of anesthesia. In one study, an increase to 80% nitrous oxide resulted in lost reflexes and complete unconsciousness following an initial period of hyperexcitability (Ridgway & McCormick, 1971). In a separate study, persistent reflexes and visual tracking at the same concentration of nitrous gas mixture was reported (Ridgway & McCormick, 1967). Further, at 80% nitrous oxide, hypoxemia and cyanosis of the mucus membranes were observed. The combination of continued presence of consciousness and inadequate oxygenation at high inspired nitrous oxide concentrations, led Ridgway and colleagues to cite the nitrous gas mixture as inadequate for major surgery in dolphins, especially as a sole anesthetic agent (Ridgway & McCormick, 1967).

Early hemodynamic studies in these anesthetized dolphins provided insights into cardiovascular function under general anesthesia. Mean arterial pressures in healthy dolphins on halothane gas anesthesia averaged 115 mmHg (normal reported as 120–140 mmHg) (Ridgway & McCormick, 1971). Dolphins on nitrous oxide‐oxygen gas anesthesia ranged between 122 and 142 mmHg (Sommer et al., 1968). Ridgway also observed that the normal, respiratory sinus arrhythmia (RSA) observed in the conscious, non‐anesthetized dolphin transitioned to a normal sinus rhythm, with heart rates between 80 and 160 bpm, after thiopental (15–25 mg/kg) administration (Ridgway et al., 1974; Ridgway & McCormick, 1971, 1967).

While Ridgway published on observational aspects of clinical anesthesia in dolphins, few comprehensive and controlled physiologic studies of anesthetized dolphins have since been conducted (McCormick, 1969; Sommer et al., 1968). Most reports are limited to single case descriptions that document individual dolphins (*Tursiops* spp.) recovering from surgical or diagnostic procedures, rather than controlled pharmacokinetic or physiologic studies on the effects of ventilation and anesthetics agents (Table 2) (Bailey, 2021; Doescher et al., 2018; Dover et al., 1999; Lee et al., 2019; Lindemann et al., 2023; Meegan et al., 2015, 2016; Meegan, Miller, et al., 2021; Ridgway, 2002; Russell et al., 2021; Schmitt et al., 2014; Tamura et al., 2017). The paucity of comprehensive, controlled anesthetic studies in bottlenose dolphins remain a hurdle in our understanding of the physiology of anesthesia in this species.

**TABLE 2:** Summary of single case reports of successful general anesthesia and recovery in dolphins (*Tursiops* spp.)

Clinical indication	Age (years)	Sex	Weight (kg)	Pre‐medication agent(s)	Induction agent(s)	Maintenance agent(s)	MAP range (mmHg)	Reversal agent(s)	Reference
Cerebral spinal fluid sampling	5	F	106	Diazepam, 0.28 mg/kg, PO	Midazolam, 0.02 mg/kg, IV	Sevoflurane	93–122	Flumazenil, 0.01 mg/kg, IM	Russell et al., 2021; Bailey, 2021
				Midazolam, 0.05 mg/kg, IM	Propofol, 2 mg/kg, IV			Flumazenil, 0.01 mg/kg, IV	
Ventral cervical abscess surgical debridement	22	F	174	Midazolam, 0.07 mg/kg, IM	Midazolam, 0.04 mg/kg, IV	Sevoflurane	44–55	Flumazenil, 0.04 mg/kg, IV	Lee et al., 2019; Meegan et al., 2015; Bailey, 2021
				Meperidine, 0.5 mg/kg, IM	Propofol, 2 mg/kg, IV			Naloxone, 0.02 mg/kg, IV	
								Naltrexone, 0.05 mg/kg, IV	
Renal biopsy, laparoscopy	27	F	150	Diazepam, 0.27 mg/kg, PO	Propofol, 3.5 mg/kg, IV	Isoflurane	N/A	Flumazenil, 0.001 mg/kg, IM	Dover et al., 1999; Bailey, 2021
				Atropine, 0.02 mg/kg, IM				Flumazenil, 0.002 mg/kg, IV	
Electroencephalography	N/A	M	140	N/A	Sodium thiopental, IV	Halothane		N/A	Ridgway, 2002
Lithotripsy	39	F	175	Midazolam, 0.07 mg/kg, IM	Midazolam, 0.06 mg/kg, IV	Sevoflurane	57–117	Flumazenil, 0.024 mg/kg, IV	Bailey, 2021
					Propofol, 3.6 mg/kg, IV				
Partial glossectomy	24	F	206	Diazepam, 0.24 mg/kg, PO	Midazolam, 0.024 mg/kg, IV	Sevoflurane	63–81	Flumazenil, 0.017 mg/kg, IV	Bailey, 2021
				Midazolam, 0.05 mg/kg, IM	Propofol, 4 mg/kg, IV			Flumazenil, 0.01 mg/kg, IM	
Corneal scleral mass	17	M	184.5	Diazepam, 0.22 mg/kg, PO	Midazolam, 0.027 mg/kg, IV	Sevoflurane	N/A	Flumazenil, 0.01 mg/kg, IV	Bailey, 2021
				Midazolam, 0.054 mg/kg, IM	Propofol, 2.87 mg/kg, IV			Edrophonium, 0.5 mg/kg, IV	
					Atracurium, 0.1 mg/kg, IV				
Mandibular sequestrum debridement	18	M	220.5	Diazepam, 0.18 mg/kg, PO	Midazolam, 0.023 mg/kg, IV	Sevoflurane	25–35	Flumazenil, 0.02*6* mg/kg, IV	Bailey, 2021
				Midazolam, 0.05 mg/kg, IM	Propofol, 1.5 mg/kg, IV			Naloxone, 0.02 mg/kg, IV	
								Naltrexone, 0.05 mg/kg, IV	
Glossectomy	44	M	215.5	Diazepam, 0.23 mg/kg, PO	Propofol, 4 mg/kg, IV	Sevoflurane	44–87	Flumazenil, 0.019 mg/kg, IV	Doescher et al., 2018; Bailey, 2021
				Midazolam, 0.08 mg/kg, IM					
Partial glossectomy	35	F	245	Diazepam, 0.25 mg/kg, PO	Midazolam, 0.02 mg/kg, IV	Sevoflurane	N/A	Flumazenil, 0.02 mg/kg, IV	Bailey, 2021
				Midazolam, 0.05 mg/kg, IM	Propofol, 2 mg/kg, IV				
Partial glossectomy	27	F	225	Diazepam, 0.25 mg/kg, PO	Midazolam, 0.02 mg/kg, IV	Sevoflurane	98–120	Flumazenil, 0.022 mg/kg, IV	Bailey, 2021
				Midazolam, 0.05 mg/kg, IM	Propofol, 1.15 mg/kg, IV				
					Atropine, 0.02 mg/kg, IM				
Partial glossectomy	20	F	190	Diazepam, 0.25 mg/kg, PO	Midazolam, 0.02 mg/kg, IV	Sevoflurane	33–65	Flumazenil, 0.025 mg/kg, IV	Bailey, 2021
				Midazolam, 0.05 mg/kg, IM	Propofol, 2.1 mg/kg, IV				
Lymphadenectomy	49	M	140	Midazolam, 0.2 mg/kg, IM	Midazolam, 0.035 mg/kg, IV	Sevoflurane	24–53	Flumazenil, 0.021 mg/kg, IV	Bailey, 2021
					Propofol, 1.5 mg/kg, IV			Naloxone, 0.04 mg/kg, IV	
Gastroscopy for foreign body retrieval	12	M	117	Midazolam, 0.1 mg/kg, IM	Midazolam, 0.042 mg/kg, IV	Sevoflurane	76–119	Flumazenil, 0.016 mg/kg, IV	Bailey, 2021
					Propofol, 2.14 mg/kg, IV			Naloxone, 0.017 mg/kg, IV	
					Atracurium, 0.2 mg/kg, IV			Naltrexone, 0.2 mg/kg, IV	
Partial glossectomy	46	F	210	Diazepam, 0.24 mg/kg, PO	Midazolam, 0.02 mg/kg, IV	Sevoflurane	N/A	Flumazenil, 0.03 mg/kg, IV	Bailey, 2021
				Midazolam, 0.02 mg/kg, IM	Propofol, 2.95 mg/kg, IV				
Oral surgery, ophthalmic examination	38	F	187.5	Midazolam, 0.08 mg/kg, IM	Midazolam, 0.02 mg/kg, IV	Sevoflurane	73–96	Flumazenil, 0.015 mg/kg, IV	Bailey, 2021
				Meperidine, 0.1 mg/kg, IM	Propofol, 2.29 mg/kg, IV			Naloxone, 0.02 mg/kg, IV	
Ophthalmic surgery	22	M	171	Diazepam, 0.1 mg/kg, PO	Midazolam, 0.02 mg/kg, IV	Sevoflurane	60–115	Flumazenil, 0.018 mg/kg, IV	Bailey, 2021
				Midazolam, 0.09 mg/kg, IM	Propofol, 3 mg/kg, IV			Naloxone, 0.03 mg/kg, IV	
				Meperidine, 0.2 mg/kg, IM					
Bronchoscopic dilatation	8	M	196	Diazepam, 0.2 mg/kg, PO	Midazolam, 0.03 mg/kg, IV	Sevoflurane	62–84	Flumazenil, 0.02 mg/kg, IV	Bailey, 2021; Meegan, Ardente, et al., 2021
				Midazolam, 0.08 mg/kg, IM	Propofol, 3 mg/kg, IV			Naloxone, 0.02 mg/kg, IV	
				Meperidine, 0.1 mg/kg, IM					
Partial glossectomy	16	M	181	Diazepam, 0.25 mg/kg, PO	Midazolam, 0.02 mg/kg, IV	Sevoflurane	39–42	Flumazenil, 0.022 mg/kg, IV	Bailey, 2021
				Midazolam, 0.05 mg/kg, IM	Propofol, 1.65 mg/kg, IV				
Tail abscess surgical debridement	14	F1	160	Midazolam, 0.075 mg/kg, IM	Propofol, 1.4 mg/kg, IV	Sevoflurane	Indirect: 36–49	Flumazenil, 0.015 mg/kg, IV	Tamura et al., 2017
				Butorphanol, 0.05 mg/kg, IM				Doxapram, 1 mg/kg, IV	
	14	F1	160	Midazolam, 0.075 mg/kg, IM	Propofol, 3.7 mg/kg, IV	Sevoflurane	Indirect: 25–95	Flumazenil, 0.015 mg/kg, IV	Tamura et al., 2017
				Butorphanol, 0.05 mg/kg, IM			Direct: 24	Doxapram, 1 mg/kg, IV	
Superficial keratectomy and cryosurgery of limbal melanoma	7.5	F2	175	Diazepam, 0.26 mg/kg, PO	Midazolam, 0.05 mg/kg, IV	Sevoflurane	N/A	Flumazenil, 0.025 mg/kg, IV	Bailey, 2021; Schmitt et al., 2014
					Propofol, 5.48 mg/kg, IV			Flumazenil, 0.025 mg/kg, IM	
	10	F2	185	Butorphanol, 0.11 mg/kg, IM	Midazolam, 0.027 mg/kg, IV	Sevoflurane	N/A	Flumazenil, 0.032 mg/kg, IV	Bailey, 2021
				Midazolam, 0.081 mg/kg, IM	Propofol, 2.4 mg/kg, IV			Edrophonium, 0.5 mg/kg, IV	
					Atracurium, 0.1 mg/kg, IV				
Dental surgery	36	M1	263	Midazolam, 0.08 mg/kg, IM	Midazolam, 0.02 mg/kg, IV	Sevoflurane	31–67	Flumazenil, 0.01 mg/kg, IV	Bailey, 2021; Meegan et al., 2016
					Propofol, 3.8 mg/kg, IV				
	37	M1	241	Midazolam, 0.08 mg/kg, IM	Midazolam, 0.02 mg/kg, IV	Sevoflurane	29–75	Flumazenil, 0.02 mg/kg, IV	Bailey, 2021
					Propofol, 4.3 mg/kg, IV			Naloxone, 0.01 mg/kg, IV	
					Meperidine, 0.4 mg/kg, IM			Naltrexone, 0.05 mg/kg, IV	
	38	M1	234	Midazolam, 0.08 mg/kg, IM	Midazolam, 0.03 mg/kg, IV	Sevoflurane	73–84	Flumazenil, 0.012 mg/kg, IV	Bailey, 2021
					Propofol, 5.5 mg/kg, IV			Naltrexone, 0.05 mg/kg, IV	
					Meperidine, 0.25 mg/kg, IM				
Corneal mass excision	15	F3	180	Diazepam, 0.25 mg/kg, PO	Propofol, 2.78 mg/kg, IV	Sevoflurane	67–91	Flumazenil, 0.014 mg/kg, IV	Bailey, 2021
				Midazolam, 0.08 mg/kg, IM	Atracurium, 0.1 mg/kg, IV			Edrophonium, 0.5 mg/kg, IV	
	16	F3	190	Diazepam, 0.21 mg/kg, PO	Midazolam, 0.026 mg/kg, IV	Sevoflurane	N/A	Flumazenil, 0.026 mg/kg, IV	Bailey, 2021
				Midazolam, 0.05 mg/kg, IM	Propofol, 2.87 mg/kg, IV			Edrophonium, 0.53 mg/kg, IV	
					Atracurium, 0.1 mg/kg, IV				
Bronchoscopy	16	M2	195	Diazepam, 0.1 mg/kg, PO	Propofol, 4 mg/kg, IV	Sevoflurane	50–64	Flumazenil, 0.15 mg/kg, IV	Bailey, 2021
				Midazolam, 0.08 mg/kg, IM	Meperidine, 0.25 mg/kg, IM			Naloxone, 0.01 mg/kg, IV	
								Naltrexone, 0.05 mg/kg, IV	
Ophthalmic surgery	17	M2	202	Diazepam, 0.2 mg/kg, PO	Midazolam, 0.03 mg/kg, IV	Sevoflurane	N/A	Flumazenil, 0.01 mg/kg, IV	Bailey, 2021
				Midazolam, 0.07 mg/kg, IM	Propofol, 2.23 mg/kg, IV			Naloxone, 0.01 mg/kg, IV	
				Meperidine, 0.25 mg/kg, IM	cis‐Atracurium, 0.1 mg/kg, IV			Naltrexone, 0.05 mg/kg, IV	
Partial glossectomy	37	F4	256	Diazepam, 0.2 mg/kg, PO	Midazolam, 0.02 mg/kg, IV	Sevoflurane	66–86	Flumazenil, 0.03 mg/kg, IV	Bailey, 2021
				Tramadol, 1.0 mg/kg, PO	Propofol, 3.05 mg/kg, IV			Naloxone, 0.023 mg/kg, IV	
Corneal repair	38	F4	243	Diazepam, 0.25 mg/kg, PO	Propofol, 1.0 mg/kg, IV	Sevoflurane		Flumazenil, 0.037 mg/kg, IV	Bailey, 2021
				Midazolam, 0.14 mg/kg, IM	Atracurium, 0.3 mg/kg, IV				
Exploratory laparoscopy	28	M	N/A	Ketamine, 1 mg/kg, IM	Midazolam, 0.02 mg/kg, IV	Sevoflurane	N/A	N/A	Lindemann et al., 2023
				Midazolam, 0.02 mg/kg, IM	Propofol, 0.6 mg/kg, IV				

## 3 MECHANISMS AND CURRENT APPROACHES TO DOLPHIN ANESTHESIA

Anesthetic and analgesic agents modulate the central nervous system (CNS) via activity on gamma‐aminobutyric acid type A (GABA_A_), N‐methyl D‐aspartate (NMDA), adrenergic alpha‐2, and opioid receptors. Ion channels, such as the family of neuronal hyperpolarization‐activated cyclic nucleotide‐gated (HCM) and two‐pore domain potassium (K2P) channels, are also known targets for anesthetic agents (Cascella et al., 2020; Pavel et al., 2020). For example, the excitatory glutamate NMDA receptor is associated with neuropathic pain and is antagonized by dissociative anesthetics like ketamine, tiletamine, and phencyclidine. The GABA_A_ receptors are targets for the CNS inhibitory effects of propofol, etomidate, alfaxalone, barbiturates, and benzodiazepines. Alpha‐2 adrenergic agonists, such as dexmedetomidine, tizanidine, and clonidine, produce effects centrally within the locus coeruleus (sedation) and dorsal horn (pain), as well as peripherally to modulate blood pressure, cardiac output, and insulin release from the pancreatic beta cells (Giovannitti Jr et al., 2015). Opioids (morphine, codeine, methadone, tramadol, meperidine, butorphanol, buprenorphine) exert their effects at central and peripheral mu, kappa, and delta opioid receptors and can cause hypotension and sinus bradycardia through depression of sinoatrial node activity. However, the most notable and often critical effects of opioids are seen as centrally‐mediated depression of the respiratory centers, whereby hypoventilation can lead to life‐threatening hypercapnia. Volatile anesthetics, like sevoflurane, isoflurane, and desflurane, depress the response to carbon dioxide in a dose‐dependent fashion and may cause sedation, in part, by inhibiting cholinergic neurotransmission in regions of the brain that regulate arousal (Vacas et al., 2013).

With these mechanisms in mind, current approaches to anesthesia of bottlenose dolphins may present several physiologic challenges for the anesthetist. The use of drugs causing and contributing to cardiopulmonary depression, as is also seen in large terrestrial mammals, is an undesired consequence leading to a variety of anesthesia‐associated co‐morbidities (Bukoski et al., 2022; Gozalo‐Marcilla et al., 2014; Menzies et al., 2016; Sage et al., 2018). Currently, no literature exists on the cardiopulmonary impacts of anesthesia protocols on dolphins. Per the experience of the authors, cardiopulmonary derangements, such as hypoventilation, ventilation‐perfusion mismatch, decreased functional residual capacity (FRC), vasodilation, and depression of cardiac contractility are often observed in anesthetized dolphins using commonly accepted anesthetic drugs (e.g., opioids, propofol, benzodiazepines, and inhalation anesthetics) and protocols (e.g., various combinations of ventilation methods and drug selection). These effects can often lead to hypoxemia, hypercapnia, hypotension, and decreased cardiac output (Berry, 2015; Haskins, 2015; Steffey et al., 2015). If not properly mitigated, these effects can impair organ perfusion, reduce oxygen delivery, and predispose the dolphin to organ injury and myopathic conditions (Bailey, 2021; Dold & Ridgway, 2014; Haulena & Schmitt, 2018). For example, decreased work of breathing and subsequent respiratory depression is a characteristic of dolphin sedation (benzodiazepines and opioids) that often demands respiratory support in the form of mechanical ventilation (Dold & Ridgway, 2014; Ridgway & McCormick, 1971, 1967). The use of propofol for induction and inhalation anesthetics for anesthesia maintenance may cause vasodilation and could lead to depressed cardiac contractility (Berry, 2015). Together, the reduced oxygen delivery to muscles could promote rhabdomyolysis, or the breakdown of skeletal muscle fibers, and lead to kidney injury from the breakdown products (e.g., myoglobin) (Bailey et al., 2012). Thus, there is a need to understand the physiologic impacts of anesthesia in dolphins, as well as develop strategies to reduce anesthesia‐associated morbidities.

As noted by Ridgway and colleagues, the out‐of‐water induction of anesthesia abates all spontaneous ventilation in dolphins (Bailey et al., 2022; McCormick & Ridgway, 2018). Mechanical ventilation is, therefore, required to prevent the pathophysiologic consequences of hypoventilation. The most employed mechanical ventilation approach in veterinary species, controlled or conventional mechanical ventilation, mirrors the normal respiratory pattern of terrestrial mammals. Dolphins, however, have an inspiratory breath‐hold respiratory phenotype with significant heart rate variation during each inspiratory‐to‐expiratory cycle (RSA) (Fahlman et al., 2020; Le‐Bert et al., 2024; McCormick, 1969). This cardiopulmonary coupling strategy may improve gas exchange in the conscious diving dolphin; however, it is completely abolished with mechanical ventilation (Fahlman et al., 2020). The uncoupling effect on efficient respiratory gas exchange under anesthesia is unknown and may be of consequence.

While mechanical ventilation is a critical feature of dolphin anesthesia, it can also promote alveolar collapse (atelectasis), leading to ventilation‐perfusion mismatching. While Nagel, Ridgway, and colleagues were able to mechanically mimic dolphin breathing using apneustic plateau ventilation (APV) through the modification of existing large animal ventilators, the availability of this mechanical ventilation strategy to dolphin veterinarians, as well as an understanding of its effect on respiratory gas exchange under anesthesia, are lacking. A ventilation strategy that maintains airway pressure above functional residual capacity (e.g., the point in the breathing cycle where alveoli are more prone to collapse) by decreasing lung volume and pressure from an elevated plateau pressure to an airway pressure at or slightly above functional residual capacity was recently described and tested on pigs, horses, and dolphins (Bratzke et al., 1998; Bukoski et al., 2022, 2024; Le‐Bert et al., 2024). In these studies, the authors compared the cardiopulmonary effects of apneustic anesthesia ventilation (AAV) and conventional mechanical ventilation (CMV) in 12 adult pigs, 10 healthy adult horses, and 10 healthy adult bottlenose dolphins. In the horse and pig studies, the authors found that AAV resulted in significantly higher respiratory system dynamic compliance (change in lung volume over the change in pleural pressure) and lower venous admixture, or physiologic shunt (Bukoski et al., 2022, 2024). In dolphins, AAV resulted in higher arterial oxygen tension and reduced alveolar dead space ventilation (Le‐Bert et al., 2024). Thus, this ventilation strategy demonstrated some physiologic advantages for cardiopulmonary function while mechanically ventilating anesthetized dolphins and warrants further investigation.

**FIGURE 1:**
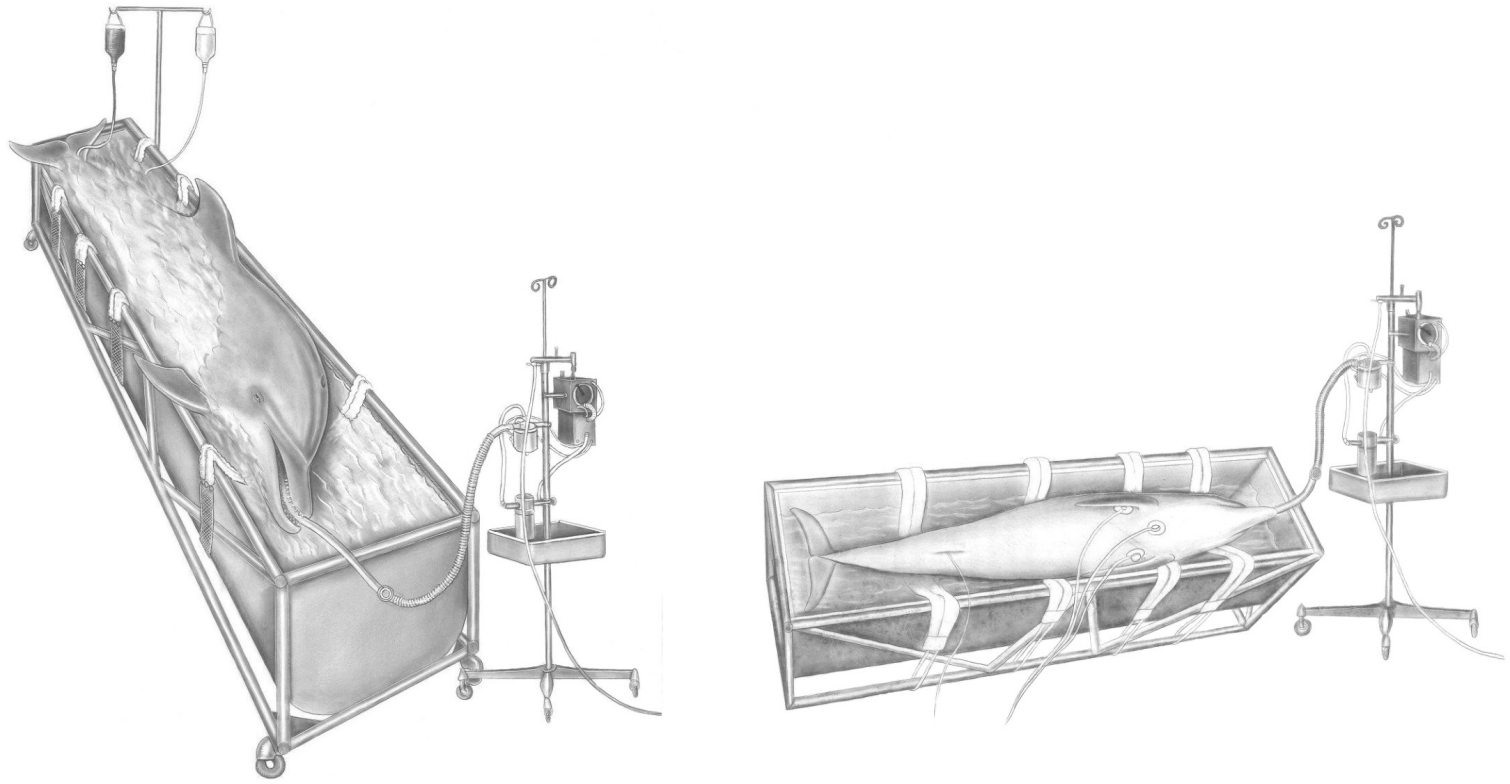
A significant challenge to general anesthesia in dolphins is the impact of gravity on a species that evolved in a buoyant ocean environment. For this reason, Ridgway would perform surgical approaches to the dolphin ear in a partially suspended state—a surgical table‐tank. The water in the surgical table‐tank was also heated to assist with thermoregulation of core body temperature (Image courtesy of the U.S. Navy's Marine Mammal Program).

Another significant challenge to the physiology of anesthesia in dolphins is the impact of gravity on a species that evolved in a buoyant ocean environment (Le‐Bert et al., 2024). When dolphins are removed from the neutrally buoyant environment, as is often necessary for medical and surgical procedures, the influence of gravity on hemodynamic variables may become an important factor (Figure 1). Resulting pressure gradients across dolphin tissues could contribute to whole body fluid shifts and blood flow redistribution when out of water for anesthetic procedures. Gravity‐induced hemodynamic shifts will be discussed in the next section and should be considered and mitigated in anesthetized dolphins when possible.

## 4 CARDIOPULMONARY ADAPTATIONS RELEVANT TO ANESTHESIA IN BOTTLENOSE DOLPHINS

While cetaceans evolved for life in diverse aquatic habitats, all cetacean species rely on intermittent surfacing to breathe air. Consequently, prolonged intervals of breath‐holding required for locomotion and foraging impact respiratory gas exchange and metabolism (Noren et al., 2012). As such, cetaceans developed specialized anatomic characteristics and physiologic adaptations which must be considered during anesthesia. Here, we expand upon select cardiovascular and pulmonary adaptations to diving and breath‐holding activities and how these adaptations may influence dolphin responses to anesthetic agents.

### 4.1 Cardiovascular system adaptations

Cetaceans exhibit unique cardiovascular system morphology and physiology to support the circulatory and metabolic requirements of a diving lifestyle. For example, a dorsal‐ventral flattening of the four‐chambered heart limits the impact of chest wall compression on ventricular filling (preload) during a dive (Ochrymowych & Lambertsen, 1984). The cetacean heart is believed to have a Purkinje fiber distribution similar to terrestrial ungulates, also referred to as a Category B, or Type 2, ventricular depolarization pattern (Calloe, 2019; Hamlin, 1970; Hamlin & Smith, 1965; Harms et al., 2013; Kinoshita et al., 2023). These larger Purkinje fibers are believed to increase signal conduction velocity from the atrioventricular node to the ventricular myocardium and may benefit the observed rapid heart rate transitions from a diving bradycardia to a resurfacing tachycardia (Storlund et al., 2021). Conversely, a recent histologic study of the dolphin heart demonstrated the Purkinje fibers actually run just below the endocardium, as seen in humans (Category A ventricular depolarization pattern), and do not extend through the myocardium as is typical of terrestrial ungulates (Kinoshita et al., 2023).

In a meta‐analysis comparing ECG morphology of 50 species of terrestrial mammals and 19 species of marine mammals, marine mammal species exhibited slower atrial (19% longer P‐wave) and ventricular depolarization (24% longer QRS interval), and faster ventricular repolarization (21% shorter QT interval) than terrestrial mammals (Storlund et al., 2021). These electrophysiologic features would suggest an effect of the larger myocardial mass of dolphins influencing the duration of the electrical signal conduction (Storlund et al., 2021). These ECG features are relevant to the physiologic monitoring of both awake and anesthetized dolphins and should, therefore, be considered in the management of perfusion states. More research into the anatomic and physiologic differences contributing to the ventricular activation pattern of dolphins and the potential impact on circulation under general anesthesia is warranted.

The vascular anatomical features of the dolphin are also important when considering the anesthetic effects on dolphin physiology. Cetacean veins and arteries are extremely specialized with respect to circulation, hemodynamics, blood storage, oxygen transport, and thermoregulation. In cetacean appendages (pectoral flippers, tail fluke, dorsal fin), arteries and veins form a complex of vessels known as periarterial venous retia (Meagher et al., 2002). Retia function as counter‐current heat exchangers to support thermoregulation (core body temperature regulation) in the thermally conductive aquatic environment. Highly specialized networks of elaborate vessels, known as *retia mirabilia* (“wonderful nets”), around the brain and spinal cord (cranial and spinal *rete mirabilis*), cervicothoracic vertebrae (cervical and thoracospinal *retia mirabilia*), gonads and eyes (cranial and ophthalmic rete mirabilis), are also key cardiovascular adaptations in cetaceans (Ballarin et al., 2018; Bonato et al., 2019; Costidis, 2012; Cozzi et al., 2017; Lillie et al., 2022; Rommel et al., 1992; Rowlands et al., 2021). These complex vascular structures consist of a single artery with many smaller branching vessels suspended among numerous small veins, giving the appearance of a vascular net or meshwork. They are the major site of blood storage in cetaceans (Bonato et al., 2019; Cozzi et al., 2017). Rete mirabilia may function in maintaining arterial blood pressure and providing adequate cerebral perfusion independent from the peripheral thermoregulatory periarterial venous retia (Lillie et al., 2022; Rowlands et al., 2021). The elaborate morphology of the retial system within the cetacean skull and vertebral canal, and its vascular connections to thoracic and abdominal cavities, likely enables hemodynamic adjustments necessary for diving (Bonato et al., 2019; Nagel et al., 1968; Rowlands et al., 2021). In the natural, neutrally buoyant condition, the lack of pressure gradients across dolphin tissues may necessitate dependence on non‐cardiac pumps to adequately circulate blood throughout the body, for example, via the dorsoventral fluke oscillations of locomotion (Lillie et al., 2022).

Aside from complex vascular retia, true veins are another interesting morphologic feature of the circulatory system in dolphins. Most dolphin veins are valve‐less, which implies the ability for bidirectional blood flow and the reliance on non‐cardiac pumps, such as muscles of locomotion and retia mirabilia, to promote adequate tissue perfusion (Costidis, 2012; Harrison & Tomlinson, 1956). This feature may be particularly important when dolphins are anesthetically immobilized, rendering non‐cardiac pumps temporarily dysfunctional.

In addition to anatomical cardiovascular adaptations advantageous for a diving lifestyle, pelagic (deep‐diving) cetaceans rely on intrinsic oxygen stores via increased hemoglobin (blood), myoglobin (muscle), neuroglobin and cytoglobin (neural), as well as increased blood volumes, to tolerate prolonged dives (Dolar et al., 1999; Noren & Williams, 2000). These features enable continued aerobic metabolism despite prolonged apnea at depth (Ponganis et al., 2011). Myoglobin concentration is 10–30 fold higher in the skeletal muscle of aquatic diving mammals versus terrestrial mammals (Kooyman et al., 1981). Increased myoglobin allows for increased oxygen storage, with subsequent release during breath‐hold underwater exercise. In general, as diving capacity increases across cetacean taxa and ecotypes, skeletal muscle myoglobin concentrations, blood volume, and hemoglobin also increase (Butler & Jones, 1997; Fago et al., 2017; Horvath et al., 1968; Noren & Williams, 2000; Remington et al., 2007; Taboy et al., 2000).

Hemoglobin also adds to whole body oxygen stores and is directly proportional to total blood volume (Snyder, 1983). The shallow‐diving bottlenose dolphin, however, does not exhibit increases in red blood cell volume, hemoglobin, or myoglobin, as is measured in the deep‐diving cetaceans (Fahlman et al., 2018). Blood volume in this coastal species is closer to terrestrial mammals at ~7.1% of body mass (Johnson et al., 2009; Ridgway & Johnston, 1966). Early studies found that hemoglobin has a higher affinity for oxygen in the small, shallow‐diving bottlenose dolphin compared to the larger, deep‐diving species (Snyder, 1983). This observation was believed to facilitate oxygen extraction from the lungs during short dives, as well as facilitating oxygen off‐loading to the tissues during deep dives when lungs are collapsed. However, more recent evidence suggests diving mammals have hemoglobin oxygenation properties similar to terrestrial mammals, and that previously observed differences in the oxy‐hemoglobin dissociation curve more likely reflect differences in red blood cell 2,3‐diphosphoglycerate (DPG) concentration (Fago et al., 2017).

Lastly, there is evidence of adaptations in neural mechanisms of cardiovascular control. Specifically, alpha‐adrenergic 2B receptors in another toothed whale, the sperm whale, exhibit protein sequence differences in the polyglutamate acid domain, which likely affect agonist‐induced phosphorylation and receptor activation (Madsen et al., 2002; Small et al., 2001). Relevant to the pharmacology of anesthetic agents, this catecholamine receptor is concentrated in the spinal cord, kidneys and vascular endothelium, and can influence sedation, analgesia, muscle relaxation, bradycardia and systemic vascular resistance. Given these sequence differences of alpha 2B adrenergic receptors, sympathomimetic drugs including alpha 2 adrenergic receptor agonists and antagonists likely exhibit differential binding affinities which may, in turn, impact cardiovascular function in anesthetized dolphins.

#### 4.1.1 Cardiovascular plasticity of the diving dolphin

The mammalian dive response consists of several key events that preserve intrinsic oxygen stores and prevent asphyxia, particularly in vital organs such as the heart and brain (Panneton, 2013). First, activation of facial trigeminal nerve reflexes during submersion induces a parasympathetic response and acetylcholine release (Berk et al., 1991; Ponganis, 2019). Acetylcholine activates heart muscarinic receptors, decreasing heart rate (bradycardia). Bradycardia lowers the chronotropic state of the heart, thereby reducing oxygen consumption, conserving oxygen stores and mitochondrial energy production. Since cardiac output is the product of heart rate and stroke volume (volume of blood ejected per heartbeat), a decrease in heart rate (bradycardia) decreases cardiac output, at least if stroke volume is unchanged. Decreased cardiac output impacts tissue oxygen delivery and thus, perfusion, and limits dive duration.

Bottlenose dolphins decrease heart rate from ~101–111 bpm to ~20–30 bpm within 1 min of water submergence (Houser, Dankiewicz‐Talmadge, et al., 2010; Williams et al., 2015, 1993). Williams and colleagues demonstrated that dive depth and exercise intensity alter the extent of bradycardia in diving dolphins (Williams et al., 2015). In that same study, dolphins at diving depth exhibited ~1.7–3.7 fold increase in heart rate over gliding values during exercise (swimming). Dive depth and duration were important modulators of that exercise response. Thus, Williams and colleagues postulated that the interplay between sympathetic and parasympathetic systems (autonomic conflict) of breath‐hold exercising at depth was responsible for the observed heart rate variability and cardiac anomalies. However, Ponganis and colleagues presented an alternative interpretation of the exercise response in diving marine mammals, de‐emphasizing the concept of autonomic conflict and proposing that instead: (1) sympathetic activation is elevated at dives even without exercise, as evidenced by maximal vasoconstriction, (2) parasympathetic cardiac vagal tone dominates over sympathetic cardiac tone in diving animals (as evidenced by bradycardia), (3) exercise modulation of heart rate during dives primarily involves reduction in parasympathetic tone versus increased sympathetic tone and, finally, (4) benign arrhythmias are common in marine mammals (Ponganis et al., 2017).

Hydrostatic pressure increases with dive depth and, thus, exerts increasing intrathoracic pressures on the dolphin cardiopulmonary system while diving. Changes in hydrostatic pressure while diving are believed to modulate chronotropic and inotropic function of the heart through its influence on pulmonary volumes, blood shunting, baroreceptors, pulmonary stretch receptors, and changes in blood gas tensions (Williams et al., 2015). Mechanical ventilation during anesthesia also increases intrathoracic pressure and can negatively impact cardiac output (Mahmood & Pinsky, 2018). In 1968, Sommer and colleagues measured stroke volume (0.4–0.8 mL/kg) and cardiac output (47–105 mL/min/kg) in four dolphins under anesthesia, noting the likely influence of mechanical ventilation and out‐of‐water experimental conditions on hemodynamic variables (Sommer et al., 1968). More recently, the availability of non‐invasive transthoracic and transesophageal echocardiography has allowed for in‐water cardiovascular evaluation of awake, spontaneously ventilating dolphins (Chetboul et al., 2012; Linnehan et al., 2021; Miedler et al., 2015; Sklansky et al., 2006). In one study, cardiac output was determined by calculating the stroke volume from the integrated blood flow velocity and the aortic cross‐sectional area at the level of the aortic valve using transthoracic echocardiography (Miedler et al., 2015). Dolphins resting at the surface had an average stroke volume of approximately 0.8 mL/kg (136 ± 19 mL) and average cardiac output of 32.2 mL/min/kg (with an average heart rate of 41 bpm). As seen in humans and other species after exercise, heart rate, stroke volume, and, therefore, cardiac output, increased significantly in the surfaced dolphins for up to 4 min following cessation of exercise activity (approximately 104%, 63%, 234%, respectively). However, no studies to date have measured stroke volume in diving dolphins. Therefore, whether exercise modulates either stroke volume or cardiac output in diving dolphins is unknown.

The cardiovascular flexibility noted in dolphins at rest or after exercise may be a critical evolutionary feature to conserve oxygen during diving by regulating pulmonary and systemic perfusion. For example, cardiovascular adjustments likely minimize blood flow to peripheral musculature during dives, while rapidly removing carbon dioxide and replenishing oxygen during the surface interval. Upon ascent, bradycardia is gradually reversed, suggesting that cardiac output and stroke volume increase (Miedler et al., 2015). Thus, a key event during the dive itself is activation of the sympathetic nervous system to elicit peripheral vasoconstriction. Peripheral vasoconstriction ensures that blood flow is shunted away from peripheral tissues and focused on critical central compartments – the brain and heart. Reversal of this vasoconstriction is essential to replenish oxygen and remove carbon dioxide from those peripheral tissues during the surface interval. Blawas and colleagues demonstrated upregulation of the arachidonate 5‐lipoxygenase (ALOX5) gene in dolphins. Since downstream leukotriene metabolites induce vasoconstriction in hypoxic rodent models and humans (Friedman et al., 1984; Ichinose et al., 2001), similar mechanisms may allow marine mammals to tolerate prolonged periods under water (Blawas, Ware, et al., 2021).

Peripheral vasoconstriction while diving increases systemic and target organ vascular resistance, and therefore, perfusion states. Vascular resistance, along with blood flow or cardiac output, determine the blood pressure which is frequently monitored during general anesthesia. In a limited number of reported out‐of‐water, non‐diving, conscious dolphins, the normal mean arterial blood pressure ranged from 120 to 140 mmHg (Ridgway & McCormick, 1971). While no studies exist specifically measuring vascular resistance in resting dolphins, genomic studies of diving marine mammals point to evolutionary pressure on endothelin pathway genes (EDN1, EDN2, EDN3, EDNRA, EDNRB). Further, there is a genetic loss of a renal amino acid transporter of arginine reabsorption (SLC6A18), which would reduce production of the vasodilatory signaling molecule nitric oxide and, thus, be a potential mechanism for promoting vasoconstriction (Hindle, 2020; Huelsmann et al., 2019; Tian et al., 2016). Additional genetic markers contributing to efficient peripheral vasoconstriction in diving dolphins are those of their intrinsic coagulation pathway. Genes encoding coagulation factor XII are absent in dolphins. Specifically, the loss of the kallikrein B1 gene protects against thrombus formation, while key coagulation factors of the extrinsic pathway required for hemostasis of damaged tissue are not lost (Huelsmann et al., 2019; Kokoye et al., 2016; Semba et al., 1998, 2000). Since vasoconstriction‐induced reduction in blood vessel diameter during diving typically increases risk for thrombus formation in other mammals, loss of these genes likely provide an evolutionary advantage to cetaceans (Haulena & Schmitt, 2018; Kokoye et al., 2016).

#### 4.1.2 Respiratory sinus arrhythmia and cardiopulmonary coupling in dolphins

Respiratory sinus arrhythmia (RSA) is the variation of heart rate with the inter‐breath interval and is seen in many species, including humans. RSA is frequently used as an index of cardiac vagal tone and overall fitness and health. Many marine mammal species, including bottlenose dolphins, demonstrate pronounced RSA during surface breathing, resulting in dramatic changes in instantaneous heart rate throughout the inter‐breath interval (Blawas, Nowacek, et al., 2021; Cauture et al., 2019; Fahlman, Miedler, et al., 2019). However, heart rate patterns during prolonged breath‐holds are similar to those resulting from RSA during extended inter‐breath intervals (Blawas, Nowacek, et al., 2021). Cardiorespiratory coupling such as RSA has been proposed in dolphins as a physiologic strategy to optimize gas exchange during the surface interval between prolonged breath‐hold dives (Fahlman et al., 2020; Fahlman, Miedler, et al., 2019; Giardino et al., 2003; Hayano et al., 1996).

### 4.2 Respiratory system adaptations

Beyond the cardiovascular system, the respiratory system in cetaceans also displays specialized physiologic and anatomic adaptations suitable for a completely aquatic life. For example, the cetacean blowhole is a result of migration of nasal passages to the top of the forehead (Berta et al., 2014). The trachea of cetaceans tends to be short and wide and holds up to 4% of total lung volume (Davenport et al., 2013; Piscitelli et al., 2013). The compliant spiraling rings of the trachea and bronchi are also unique; they retain enough rigidity to remain patent, but enough flexibility to withstand the extreme compression experienced during a dive (Denk et al., 2020; Moore et al., 2013).

Cetacean lungs are unilobular and lie dorsal to the heart while enclosed within a complete mediastinum. The lungs exhibit morphological advantages essential for the explosive, intermittent ventilation observed during surfacing intervals. An abundance of elastic fibers, high pulmonary compliance, collateral ventilation adaptations, and cartilaginous reinforcement of bronchi and bronchioles, allow cetacean alveoli to undergo compression and collapse at extreme hydrostatic pressures (Piscitelli et al., 2013).

In larger airways and the extra‐pulmonary bronchi, smooth muscle replaces elastic layers. The presence of structurally reinforced airways allows for the accumulation of air at high pressures within the dead space when non‐reinforced alveoli would collapse. Alveolar collapse could protect against nitrogen gas absorption or gas emboli when ascending from a dive. Some investigators hypothesize that these attributes, along with evidence of hypoxic pulmonary vasodilation rather than vasoconstriction, and intrapulmonary arteriovenous shunts, enable functional pulmonary shunting at any depth in cetaceans; this ability for pulmonary shunts may not be fully dependent on hydrostatic compression (Garcia Parraga et al., 2018). Thus, even with alveolar collapse, extensive collateral ventilation plus hypoxia‐induced vasodilation may enable continued gas exchange in upper parts of the lung (Gompelmann et al., 2013).

#### 4.2.1 Mechanics of breathing in the dolphin

The inspiratory, breath‐holding breathing pattern of cetaceans is an essential feature required for underwater feeding activities. The defining characteristic of volitional breathing in bottlenose dolphins is a rapid exhalation of large air volumes (~90%–95% of total lung capacity) through the blowhole within approximately 0.26–0.5 s, followed by a slower inspiratory phase ending with blowhole closure. Thus, the respiratory system maintains in an inflated state with the inspired air until the next respiratory cycle (Cotten et al., 2008; Fahlman et al., 2015, 2017; Fahlman, Brodsky, et al., 2019; Piscitelli et al., 2013; Ridgway, 1972).

Existing physiologic measures of lung function and mechanics should be considered when applying mechanical ventilation during general anesthesia of dolphin patients (Table 3). Dolphin airway (alveoli) opening pressures, for example, are 21–25 cm H_2_O, with maximum lung volume achieved at pressures around 30 cm H_2_O (Piscitelli et al., 2010). Tidal volumes reported in the literature range from 15 to 22 mL/kg for dolphins resting at the surface (terrestrial mammals are about 7.7 mL/kg), while breathing frequency can be significantly variable, averaging between 0.9 and 3.6 breaths/minute in the resting dolphin (Fahlman et al., 2015; Mortola & Seguin, 2009; Piscitelli et al., 2013; Ridgway, 1972). Total lung capacity in bottlenose dolphins is reported to be between 40 and 138 mL/kg (lung mass ~2.7% of total body mass); however, some of these measurements were acquired from excised lungs, possibly contributing to the wide ranges reported (Fahlman et al., 2015, 2017; Mortola & Seguin, 2009; Piscitelli et al., 2010, 2013).

**TABLE 3:** Summary of cardiopulmonary variables relevant to perfusion adaptations of awake and anesthetized bottlenose dolphins (*Tursiops truncatus*).

	Reported values	References
Cardiovascular variables		
Heart rate (beats/min)	20–140^a^	Williams et al., 2015; Williams et al., 1993; Ponganis et al., 2017
Mean arterial pressure (mmHg)	120–142^b^	Sommer et al., 1968; Ridgway & McCormick, 1971
Cardiac index (mL/min/kg)	47–105^c^	Sommer et al., 1968
	18.2–49.5^d^	Miedler et al., 2015
Stroke volume index (mL/beat/kg)	0.4–0.8^c^	Sommer et al., 1968
	0.5–0.9^d^	Miedler et al., 2015
Blood volume (mL/kg)	65–83 (mean 71)	Johnson et al., 2009; Ridgway & Johnston, 1966
Hemoglobin (g/dL)	13.2–15.3 (mean 14.4)	Ridgway & Johnston, 1966
Oxygen‐carrying capacity, blood (mL O_2_/dL)^e^	17.7–20.5 (mean 19.3)	Ridgway & Johnston, 1966
Myoglobin (g 100/g muscle)	2.5–3.5	Kooyman & Ponganis, 1997; Dolar et al., 1999; Ridgway & Johnston, 1966
Oxygen‐carrying capacity, muscle		
Muscle mass (mL O_2_/kg muscle)^e^	33.5–46.9	Kooyman & Ponganis, 1997
Body mass (mL O_2_/kg body mass)	13.3	Pabst D, et al., 1999
Total oxygen (mL O_2_/kg)	29–36	Pabst D, et al., 1999; Kooyman & Ponganis, 1997; Noren & Williams, 2000
Pulmonary variables		
Breathing frequency (breaths/min)	0.9–3.6	Piscitelli et al., 2010; Piscitelli et al., 2013; Fahlman et al., 2015; Fahlman et al., 2017
Inspiratory phase time (ms)	430–660	Fahlman et al., 2015; Fahlman et al., 2017; Fahlman, Brodsky, et al., 2019
Expiratory phase time (ms)	260–500	Piscitelli et al., 2013; Fahlman et al., 2015; Fahlman et al., 2017; Fahlman, Brodsky, et al., 2019
Inspiratory flow rate (L/s)	9.8–20.2	Fahlman et al., 2015
Expiratory flow rate (L/s)	16.5–37.5	Fahlman et al., 2015
Tidal volume (mL/kg)	15–22	Fahlman et al., 2015; Fahlman et al., 2017; Fahlman, Miedler, et al., 2019; Mortola & Seguin, 2009
Total lung capacity (mL/kg)	40–138	Piscitelli et al., 2010; Piscitelli et al., 2013; Fahlman et al., 2015; Fahlman et al., 2017; Mortola & Seguin, 2009
Airway (alveolar) opening pressure (cm H_2_O)	21–25	Piscitelli et al., 2010; Piscitelli et al., 2013
Peak airway pressure (cm H_2_O)	30	Piscitelli et al., 2010; Piscitelli et al., 2013
Dynamic compliance (L/cm H_2_O)	0.37 ± 0.04^f^	Fahlman et al., 2015

^a^ Normal respiratory sinus arrhythmia accounts for heart rate variability in adult bottlenose dolphins at the surface and during diving conditions.

^b^ Mean arterial pressures obtained in awake as well as anesthetized out‐of‐water dolphins. No significant differences were found between both conditions, therefore, data was pooled.

^c^ Variable data obtained in anesthetized out‐of‐water dolphins via indocyanine green dye‐dilution curves (*n* = 4).

^d^ Variable data obtained in awake in‐water dolphins via echocardiogram measurements (*n* = 14).

^e^ Calculated value, that is, oxygen capacity is calculated from measured [Mb] or [Hb] assuming a conversion factor of 1.34 mL O_2_.

^f^ Estimated as tidal volume divided by tidal change in transpulmonary pressure measured using an esophageal pressure catheter.

#### 4.2.2 Control of breathing in dolphins

Breathing in mammals is a complex physiologic process involving respiratory neurons of the brainstem. Brainstem respiratory neurons generate the breathing rhythm, and then modulate that rhythm through complex feedback from chemoreceptors (both central and peripheral) and mechanoreceptors. The fundamental breathing signal is then exposed to breath pattern formation, crafting the detailed spatiotemporal distribution to the different respiratory muscles that generate a breath, as well as major inputs to breathing from cortical areas governing the volitional control of breathing (Ashhad et al., 2022).

The central and peripheral chemoreceptors sense carbon dioxide in the blood and neural tissue, while only the peripheral chemoreceptors sense changes in oxygen in the blood, triggering chemoreflexes that manifest as increased breathing (Guyenet & Bayliss, 2015; McCulloch et al., 1997). These chemoreceptors normally operate in the manner of a negative feedback loop to preserve oxygen and carbon dioxide homeostasis.

In 1969, McCormick evaluated the effects of mixed inspired gases on peripheral and central chemoreceptor responses of conscious dolphins (McCormick, 1969). In this study, increasing the inspired carbon dioxide to 5% (with normal oxygen levels) induced a ventilatory response characterized by an increase in respiratory rate. This ventilatory response persisted, even when inspired oxygen concentrations were artificially increased to 40%. Conversely, when inspired oxygen was reduced to 10%, respiratory rate again increased, consistent with a hypoxic chemoreflex elicited by peripheral (carotid body) chemoreceptors. Both responses in dolphins are consistent with normal mammalian peripheral and central chemoreflexes in response to increased inspired carbon dioxide or decreased inspired oxygen concentrations at sea level.

During diving behavior, these responses appear to be reversibly blunted. Pelagic marine mammals appear to tolerate arterial oxygen tensions (p_a_O_2_) as low as 12 mmHg and venous oxygen tensions (p_v_O_2_) as low as 3 mmHg during free diving conditions (Meir et al., 2009; Ponganis et al., 2011). Dolphin venous oxygen tensions (p_v_O_2_) during voluntary breath holds at the surface were measured as low as 18–20 mmHg (Williams et al., 1999). It was proposed by Stephenson that dis‐facilitation, rather than overt inhibition of ventilation, during diving occurs due to profound hypocapnia, decreasing the drive to breathe below the CO_2_ apneic threshold during long dives (Stephenson, 2005). In Stephenson's model, the most important factor leading to dis‐facilitation of respiratory drive occurs during the surface interval between dives. Stephenson speculated that surface breathing drastically decreases arterial carbon dioxide (<30 mmHg) and increases arterial oxygen (~120 mmHg), minimizing all chemoreflex drive to breathing (i.e., dis‐facilitation). Similar to terrestrial mammals, the drive to breathe in pelagic marine mammals is likely influenced more by central chemoreceptor responses to carbon dioxide versus peripheral chemoreceptor response to decreased oxygen levels (Stephenson, 2005). This may be an important consideration when allowing for permissive hypercapnia during emergence from general anesthesia.

A summary of the relevant, published cardiopulmonary measurements, obtained in both unanesthetized and anesthetized bottlenose dolphins is provided in Table 3 for reference.

## 5 CONCLUDING REMARKS

The ability to promote an anesthetic state in dolphins with minimal cardiopulmonary derangements is critical to reduce anesthesia‐associated morbidities and improve clinical outcomes. Current practices in dolphin anesthesia involve use of agents that cause respiratory depression and undermine cardiac function. While many of these effects can be mitigated with controlled mechanical ventilation and chronotropic, inotropic and vasopressive agents, these interventions are often complex, requiring specialized equipment and drug delivery methods. As such, limited peer‐reviewed publications exist on the pharmacokinetics of anesthetic drugs in bottlenose dolphins. Therefore, extrapolation of drug dosages from terrestrial species and human medicine is often performed. As evidenced from the distinct features of dolphin cardiopulmonary physiology, as well as genomic and receptor differences that could impact binding and metabolism of anesthetic agents as outlined above, the mechanisms of anesthesia may not translate from comparative species. Consideration of the specialized structure and function of the cardiopulmonary system of dolphins should, therefore, guide anesthetic practices to minimize its effects on cardiopulmonary depression and promote hemodynamic stability in the anesthetized dolphin. For example, investigations into partial and total intravenous anesthetic techniques that would limit reliance on inhalation anesthetics may provide cardio‐pulmonary sparing effects and improve perfusion states. Additionally, evaluation of mixed gas ventilation approaches, such as lower inspired oxygen concentrations, may reduce morbidities associated with absorption atelectasis. And finally, limiting the gravitational effects on dolphin circulation by investigating buoyant materials may improve hemodynamics and thus, perfusion, under general anesthesia.

Though we recognize the need to improve anesthesia protocols in dolphins, a significant hurdle in the advancement of dolphin anesthesiology is the relative lack of dolphins under human care around the world. The low availability of study subjects and resource expertise makes controlled basic physiologic and pharmacologic studies difficult to complete. Bottlenose dolphins considered ‘charismatic megafauna,’ are not routinely subjected to risky and complex biomedical studies. A greater understanding of dolphin physiology will continue to contribute to improvements in anesthesia protocols and medical management and may even inspire advances in human biomedical research and health care. However, in the West, dolphins are not managed as laboratory animals and thus, opportunities for controlled biomedical studies will remain limited. Often these opportunities present by observations incidental to emergent or urgent clinical interventions and dependent on gradually evolving approaches to medical management of healthy dolphins among larger holders of this species. For these reasons, it is of utmost importance these and smaller holding institutions maintain a network of scientists and collaborators with expertise in cetacean medicine, physiology, and anesthesiology. Only with these collaborations can the practice and discipline of dolphin anesthesiology eliminate the hurdle of inexperience and advance anesthetic practices for the improvement of cetacean care around the world.

## FUNDING INFORMATION

Funding for this manuscript was provided to C. Le‐Bert through the Naval Information Warfare Center Pacific, Naval Innovative Science and Engineering (NISE) Program, P‐NISE‐AR‐23‐230,205;76,725.
